# Resistance to ROS1 Inhibition Mediated by EGFR Pathway Activation in Non-Small Cell Lung Cancer

**DOI:** 10.1371/journal.pone.0082236

**Published:** 2013-12-13

**Authors:** Kurtis D. Davies, Sakshi Mahale, David P. Astling, Dara L. Aisner, Anh T. Le, Trista K. Hinz, Aria Vaishnavi, Paul A. Bunn, Lynn E. Heasley, Aik-Choon Tan, D. Ross Camidge, Marileila Varella-Garcia, Robert C. Doebele

**Affiliations:** 1 Department of Medicine, Division of Medical Oncology, University of Colorado - Anschutz Medical Campus, Aurora, Colorado, United States of America; 2 Department of Pathology, University of Colorado - Anschutz Medical Campus, Aurora, Colorado, United States of America; 3 Department of Craniofacial Biology, University of Colorado - Anschutz Medical Campus, Aurora, Colorado, United States of America; H. Lee Moffitt Cancer Center & Research Institute, United States of America

## Abstract

The targeting of oncogenic ‘driver’ kinases with small molecule inhibitors has proven to be a highly effective therapeutic strategy in selected non-small cell lung cancer (NSCLC) patients. However, acquired resistance to targeted therapies invariably arises and is a major limitation to patient care. ROS1 fusion proteins are a recently described class of oncogenic driver, and NSCLC patients that express these fusions generally respond well to ROS1-targeted therapy. In this study, we sought to determine mechanisms of acquired resistance to ROS1 inhibition. To accomplish this, we analyzed tumor samples from a patient who initially responded to the ROS1 inhibitor crizotinib but eventually developed acquired resistance. In addition, we generated a ROS1 inhibition-resistant derivative of the initially sensitive NSCLC cell line HCC78. Previously described mechanisms of acquired resistance to tyrosine kinase inhibitors including target kinase-domain mutation, target copy number gain, epithelial-mesenchymal transition, and conversion to small cell lung cancer histology were found to not underlie resistance in the patient sample or resistant cell line. However, we did observe a switch in the control of growth and survival signaling pathways from ROS1 to EGFR in the resistant cell line. As a result of this switch, ROS1 inhibition-resistant HCC78 cells became sensitive to EGFR inhibition, an effect that was enhanced by co-treatment with a ROS1 inhibitor. Our results suggest that co-inhibition of ROS1 and EGFR may be an effective strategy to combat resistance to targeted therapy in some ROS1 fusion-positive NSCLC patients.

## Introduction

Lung cancer, of which approximately 80–85% can be categorized as non-small cell lung cancer (NSCLC), is the leading cause of cancer related mortality in the world [1]. Recently, it has become clear that NSCLC is a heterogeneous disease that can be largely subdivided based on genetic alterations that create dominant driver oncogenes [2]. NSCLC tumor cells are often ‘addicted’ to these activated oncogenes, such that inhibition of their activity blocks proliferative and pro-survival cellular signaling, ultimately leading to growth arrest and/or cell death. Importantly, many of the oncogenic drivers discovered to date are activated kinases that can be targeted by small molecule inhibitors. Gefitinib and erlotinib treatment of NSCLC patients harboring *EGFR* activating mutations and crizotinib treatment of NSCLC patients harboring activating *ALK* rearrangements are successful examples of this strategy [3], [4]. Treatment with these kinase inhibitor drugs results in improved efficacy and has more tolerable side effects compared to standard chemotherapies in patients who are pre-screened for the activating genetic alterations [5], [6], [7].

Despite the initial efficacy of gefitinib, erlotinib, and crizotinib in selected NSCLC patients, acquired resistance invariably arises, typically in less than one year. At the cellular level, this resistance occurs by several mechanisms. The first of these is mutation of the target kinase domain that reduces the ability of the drug to inhibit the kinase. For example, the T790M mutation, termed the ‘gatekeeper’ mutation, reduces the ability of EGFR inhibitors to outcompete ATP binding to EGFR [8]. This mutation (along with other far less frequent resistance-associated mutations) is found in cell line models of resistance and in approximately 50% of patients who develop acquired resistance to EGFR inhibitor therapy [9], [10], [11]. The analogous gatekeeper position on ALK, L1196, is similarly found to be mutated in ALK fusion-positive lung cancer at the time of resistance to crizotinib and in resistant cell line models, as are several other amino acids for which mutation also reduces the ability of the drug to inhibit the kinase [12], [13], [14], [15], [16]. The second mechanism of resistance is amplification of the target kinase. In theory, an increase in the amount of kinase that is expressed by the cell can reduce the ability of the drug to saturate the target. Amplification of *ALK* fusions has been demonstrated in resistant cells and patients who have developed resistance [13], [14], [16]. In addition, amplification of *EGFR* has been correlated with resistance in *EGFR* mutant lung cancer, although in the majority of cases the amplified allele harbors the T790M mutation [17], [18]. Another major mechanism of resistance is activation of alternative signaling components. In this case, proteins that are downstream from or that function in parallel to the target kinase become activated and subvert the reliance on the target kinase to exclusively stimulate proliferative and pro-survival signaling. MET, PI3K, BRAF, IGF-1R, FGFR1, MEK, and ERK1/2 activation have all been observed in EGFR inhibitor-resistant *EGFR* mutant disease and/or cell line models [18], [19], [20], [21], [22], [23], [24]. Activation or amplification of EGFR, KRAS, and KIT have similarly been observed in crizotinib-resistant *ALK* rearranged patients and cell lines [14], [15], [16], [25]. Furthermore, a recent study demonstrated that exposure to common growth factors is sufficient to induce resistance to targeted therapies in cancer cell lines from a variety of genetic backgrounds, and this effect correlated with a rescue from drug-induced AKT and ERK inactivation [26]. Finally, histological and morphological changes have been demonstrated to correlate with resistance. Specifically, conversion to small cell lung cancer histology and epithelial-mesenchymal transition (EMT) have been observed in *EGFR* mutant patients and cell lines resistant to EGFR inhibitors [11], [18]. The mechanistic bases behind these changes are not completely understood.

*ROS1* is a receptor tyrosine kinase that is closely related to *ALK*, and, like *ALK*, it undergoes genomic rearrangement that creates fusion proteins in NSCLC and other cancers [27]. It is well established that these fusion proteins act as oncogenic drivers and that ROS1 inhibition is anti-proliferative in cells that express ROS1 fusions [28]. In addition, crizotinib, which has activity against ROS1, is demonstrating efficacy in *ROS1* fusion-positive NSCLC patients in a phase I trial [29]. Thus, considering the success of crizotinib in *ALK* fusion-positive NSCLC, it appears that ROS1 targeted therapy will likely soon be the standard of care for this patient population. However, based on the experiences with other kinase inhibitors in lung cancer, it is fully expected that acquired resistance to ROS1 inhibition will occur, and this will ultimately limit the treatment options for these patients. In support of this, a recent study reported a clinical case of a *ROS1* rearrangement-positive patient who developed resistance to crizotinib following an initial response [30]. The patient was found to have undergone a mutation in the *ROS1* kinase domain that interfered with drug binding [30]. In this study, we sought to determine mechanisms of resistance to ROS1 inhibition. This was accomplished using clinical samples from a patient that became resistant to crizotinib and a ROS1 inhibition-resistant derivative of the *ROS1* rearranged NSCLC cell line HCC78.

## Results

**Figure 1:**
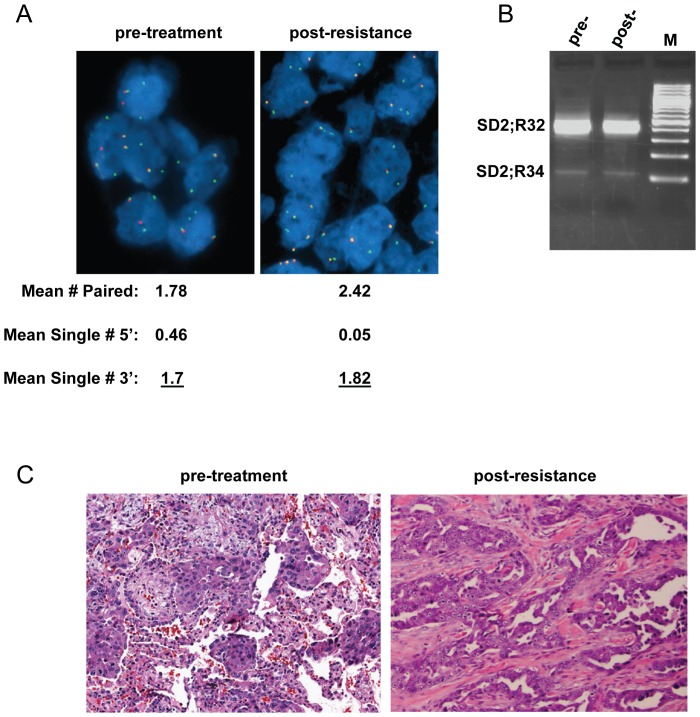
Crizotinib-resistant patient sample does not indicate *ROS1* gene amplification or histologic change. (A) Pre-treatment and post-resistance patient samples analyzed by break-apart FISH assay for *ROS1*. Red probes are to the 5′ region of ROS1 and green probes to the 3′ region. Values represent the mean number of signals per cell. The single 3′ signal (values underlined) is indicative of the *ROS1* fusion gene copy number. (B) RT-PCR, using primers to *SDC4* and *ROS1* that span the fusion point, performed on pre-treatment and post-resistance tumor samples. SD2;R32 is the ‘long’ variant (fusion of *SDC4* exon 2 to *ROS1* exon 32) and SD2;R34 is the ‘short’ variant (fusion of *SDC4* exon 2 to *ROS1* exon 34). (C) Hematoxylin and eosin staining of pre-treatment and post-resistance patient samples.

We previously reported the identification of a NSCLC patient who expressed the *SDC4-ROS1* fusion and the successful treatment of this patient with crizotinib [28]. The patient experienced 57% tumor shrinkage after two 28-day treatment cycles. However, despite continuous therapy, evidence of disease progression was discovered approximately 18 weeks after the start of treatment. At this time, an excisional biopsy of the progressing tumor was taken. Targeted sequencing of this resistant biopsy sample revealed that, similar to the pre-treatment biopsy, the entire *ROS1* kinase domain was wild-type (WT), meaning that *ROS1* kinase domain mutation was not the mechanism of resistance (Table 1). SNaPshot analysis also indicated WT status of commonly mutated residues in several other known oncogenes (including *EGFR*, *KRAS*, and *BRAF*) in both the pre-treatment and post-resistance samples (Table 1). We then examined copy number gain of the *ROS1* fusion gene as a potential mechanism of resistance. This was accomplished using fluorescence *in situ* hybridization (FISH) with probes to both the 5′ and 3′ regions of the gene. The mean single 3′ signal (representative of the fusion gene) number per tumor cell was 1.7 in the pre-treatment sample and 1.82 in the post-resistance sample, a non-significant difference (Figure 1A). This finding suggested that copy number gain was not the mechanism of resistance in this patient’s tumor. The post-resistance sample did reveal a loss in the single 5′ signal; however this is not expected to be functionally significant (Figure 1A). We verified that the fusion gene was being expressed at the time of resistance, and that the ratio of long (*SDC4* exon 2 fused to *ROS1* exon 32 (SD2;R32)) to short (*SDC4* exon 2 fused to *ROS1* exon 34 (SD2;R34)) variants was similar to the pre-treatment sample (Figure 1B). Morphologic examination of the specimen taken at resistance demonstrated plump epithelioid cells with a large nuclear to cytoplasmic ratio, prominent nucleoli, and eosinophilic cytoplasm, similar to the pre-treatment biopsy (Figure 1C). These findings suggested that small cell transformation had not occurred. Finally, no morphologic evidence of EMT, such as cellular spindling, was observed, and the biopsy taken at resistance demonstrated negative immunohistochemical staining for vimentin, an EMT marker (Figure S1). The lack of evidence for these common resistance mechanisms was suggestive of upregulation of alterative signaling as the underlying mechanism of resistance to crizotinib in this patient.

**Table 1:** 

	pre-treatmentpatient sample	post-resistancepatient sample	parental HCC78	HCC78-TR
***ROS1*** ** kinase domain**	WT	WT	WT	WT
***EGFR***	WT	WT	WT	WT
***KRAS***	WT	WT	WT	WT
***BRAF***	WT	WT	WT	WT

**Figure 2:**
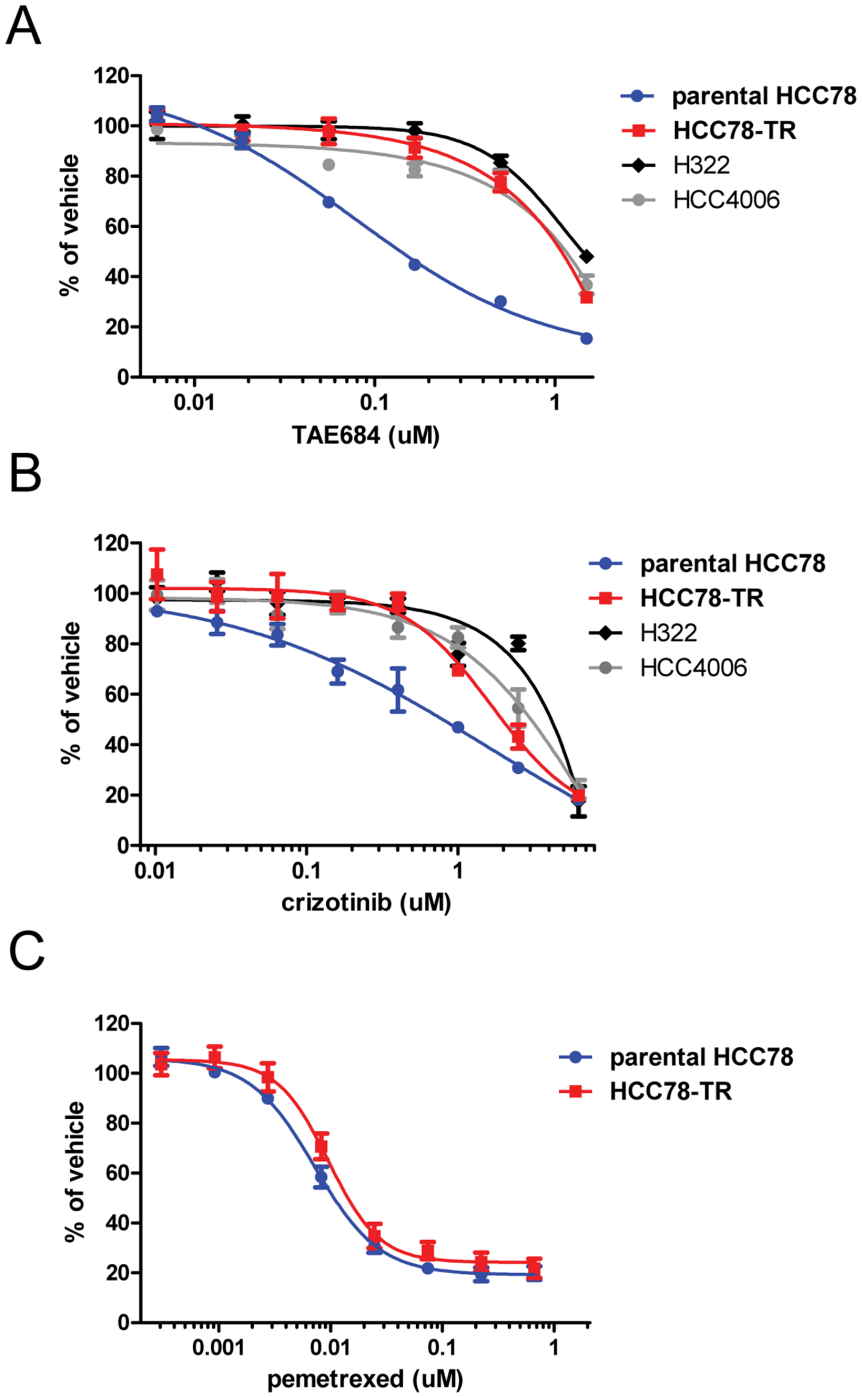
HCC78-TR cells are resistant to ROS1 inhibition. Cells were treated with TAE684 (A), crizotinib (B), or pemetrexed (C) as single-agents for 3 days and then analyzed by MTS assay. Values represent the mean ± SEM (n = 3–7). Calculated IC_50_ values for TAE684: parental HCC78 = 0.14 µM, HCC78-TR = 1.09 µM, H322 = 1.42 µM, and HCC4006 = 1.15 µM. Calculated IC_50_ values for crizotinib: parental HCC78 = 0.79 µM, HCC78-TR = 1.95 µM, H322 = 4.13 µM, and HCC4006 = 3.03 µM. Calculated IC_50_ values for pemetrexed: parental HCC78 = 11****nM and HCC78-TR = 14****nM. HCC78-TR cells were significantly less sensitive than parental HCC78 cells to TAE684 (p<0.000005) and crizotinib (p<0.05) but not pemetrexed (p>0.05) as determined by student’s paired t-test.

In order to create a cell-line model of resistance to ROS1 inhibition, we chronically treated the *SLC34A2-ROS1* expressing NSCLC cell line HCC78 with increasing concentrations of the ROS1 inhibitor TAE684. This method has been used previously to create resistant models for both EGFR inhibitors in *EGFR* mutant cells and crizotinib in *ALK* rearranged cells, and the mechanisms observed in these models have correlated with what is observed in patients [13], [15], [19]. We chose to use TAE684 (a non-clinical compound) over crizotinib (a drug with clinical activity against ROS1) because crizotinib has a relatively high IC_50_ in HCC78 cells and only a very narrow window exits between the IC_50_ and off-target activities of the drug [28], [31]. In other words, by using TAE684 to make the cells resistant to ROS1 inhibition instead of crizotinib, we were able to ensure a more complete inhibition of the ROS1 fusion protein at doses that did not exhibit off-target anti-proliferative effects. After approximately 4 months of culture in increasing concentrations, the resistant derivative of the HCC78 line, which we termed HCC78-TR, was able to proliferate normally in 500****nM TAE684. Initial attempts to increase the dose in culture further were unsuccessful, so 500****nM was considered a maximum and the cells were continuously cultured in this concentration of drug. When the sensitivity of these cells to TAE684 was analyzed in proliferation assays, it was determined that the IC_50_ of TAE684 was greater than 1 µM (Figure 2A). This is similar to other NSCLC cell lines that do not contain ALK or ROS1 fusions (H322 and HCC4006), suggesting that the anti-proliferative effects in this range are likely due to off-target activities. HCC78-TR cells were also less sensitive to crizotinib, and again, the level of sensitivity was more similar to cells that do not express ALK or ROS1 fusions (Figure 2B). However, the desensitization was specific to ROS1 inhibition, as the HCC78-TR cells retained their sensitivity to pemetrexed, an FDA-approved chemotherapy for non-squamous lung cancer (Figure 2C). The resistance to ROS1 inhibition was not dependent on the continuous culturing in the presence of 500****nM TAE684, because cells that were taken out of drug remained resistant for up to 6 months and 47 passages (Figure S2).

**Figure 3:**
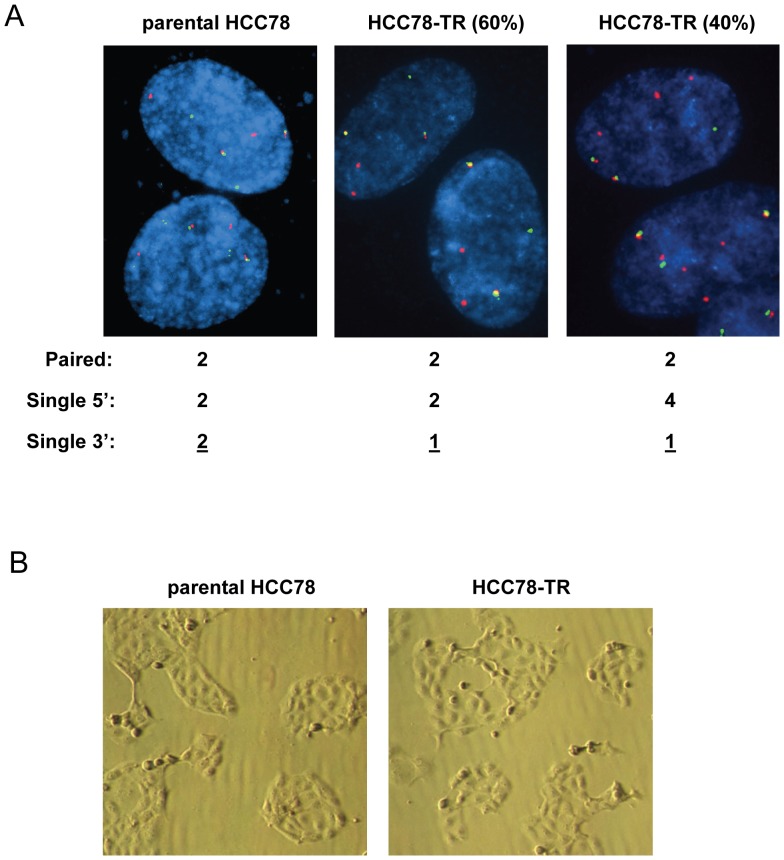
HCC78-TR cells do not exhibit *ROS1* gene amplification or morphological changes compared to parental HCC78 cells. (A) Parental HCC78 and HCC78-TR cells analyzed by break-apart FISH assay for *ROS1*. Red probes are to the 5′ region of ROS1 and green probes to the 3′ region. Values represent the number of signals per cell. The single 3′ signal (values underlined) is indicative of the *ROS1* fusion gene copy number. In the HCC78-TR line, two populations existed that differed based on the number of 5′ signals detected. (B) Representative bright field images of parental HCC78 and HCC78-TR cells.

Sequencing of the parental HCC78 and HCC78-TR cells indicated that the kinase domain of *ROS1* was WT for both lines, suggesting that *ROS1* mutation was not responsible for the resistance to ROS1 inhibition (Table 1). *EGFR*, *KRAS*, and *BRAF* were also found to be WT in both cell lines (Table 1). FISH analysis demonstrated that the HCC78-TR cells lost 1 copy of the *ROS1* fusion gene as compared to the parental line (1 copy vs. 2 copies, respectively), suggesting that copy number gain of the fusion gene was not the mechanism of resistance (Figure 3A). As a result of the genomic loss of 1 copy of the fusion gene, less *SLC34A2-ROS1* mRNA was expressed in the HCC78-TR cells (Figure S3). Although the significance of the reduced fusion gene expression is unclear, forced expression of an activated ROS1 fusion protein (SDC4-ROS1) in the HCC78-TR cells did not re-sensitize them to TAE684 (Figure S4). Approximately 40% of the HCC78-TR cells displayed an increase in the number of copies of the 5′ region of *ROS1* (from 2 copies to 4 copies), although this is not expected to be functionally significant as the 5′ region does not exhibit kinase activity (Figure 3A). In addition, the morphology of the HCC78-TR cells did not visually differ from that of the parental HCC78 line, suggesting that conversion to a small cell lung cancer morphology did not occur (Figure 3B). By mRNA quantitation, *CDH1* levels were similar between the two cell lines and *VIM* levels were decreased 3-fold in the HCC78-TR line (Figure S5). These results suggest that EMT was not the mechanism of resistance. Finally, we did not observe significant increases in mRNA expression of any of the ATP-binding cassette transporter family genes in the HCC78-TR cells, suggesting that enhanced drug efflux most likely did not account for the resistance to ROS1 inhibition (Table S1).

**Figure 4:**
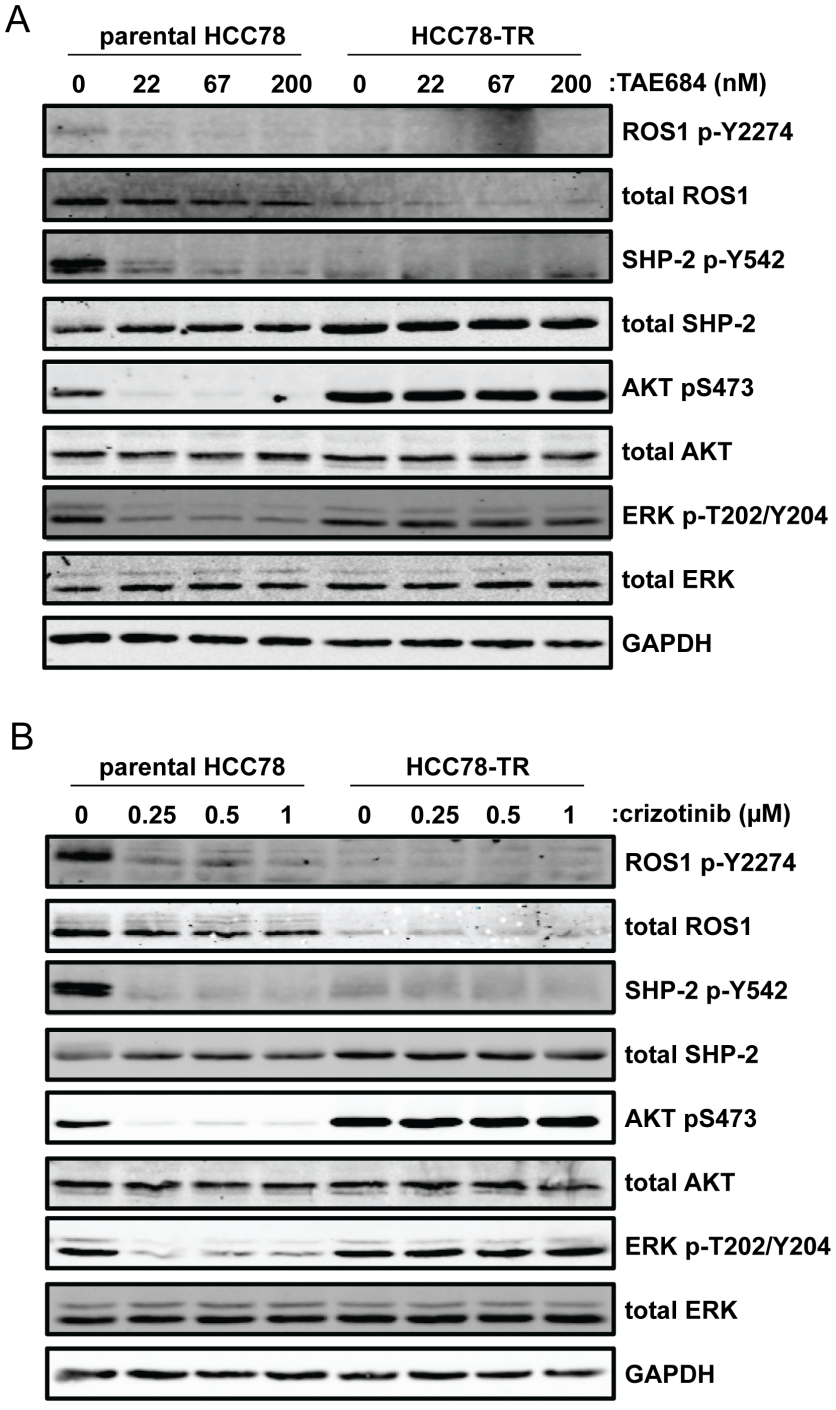
Growth and survival signaling pathway activation is refractory to ROS1 inhibition in HCC78-TR cells. Cells were treated with TAE684 (A) or crizotinib (B) for 4 hours. Lysates of the cells were then analyzed by Western blot using the indicated antibodies.

We then asked whether the resistance to ROS1 inhibition could be due to changes in downstream cellular signaling. Parental HCC78 and HCC78-TR cells were treated with a dose-range of TAE684 for 4 hours and then cell lysates were analyzed by western blot. Similar to what we have previously reported, TAE684 treatment reduced ROS1 autophosphorylation and activating phosphorylation of SHP-2, AKT, and ERK1/2 in the parental HCC78 cells (Figure 4A) [28]. As predicted from the genomic copy number loss of the *ROS1* fusion gene and the reduced mRNA expression, the HCC78-TR cells expressed less of the fusion protein than the parental line and ROS1 autophosphorylation could not be detected. The reduction in the amount of ROS1 fusion protein correlated with a reduction in the basal phosphorylation of SHP-2. However, despite the fusion protein loss, basal levels of phosphorylated ERK1/2 were similar to the parental line and basal levels of phosphorylated AKT were greater than in the parental line. Importantly, TAE684 treatment did not result in de-phosphorylation of AKT or ERK1/2 in the resistant cells. Similar results were observed with crizotinib treatment (Figure 4B).

**Figure 5:**
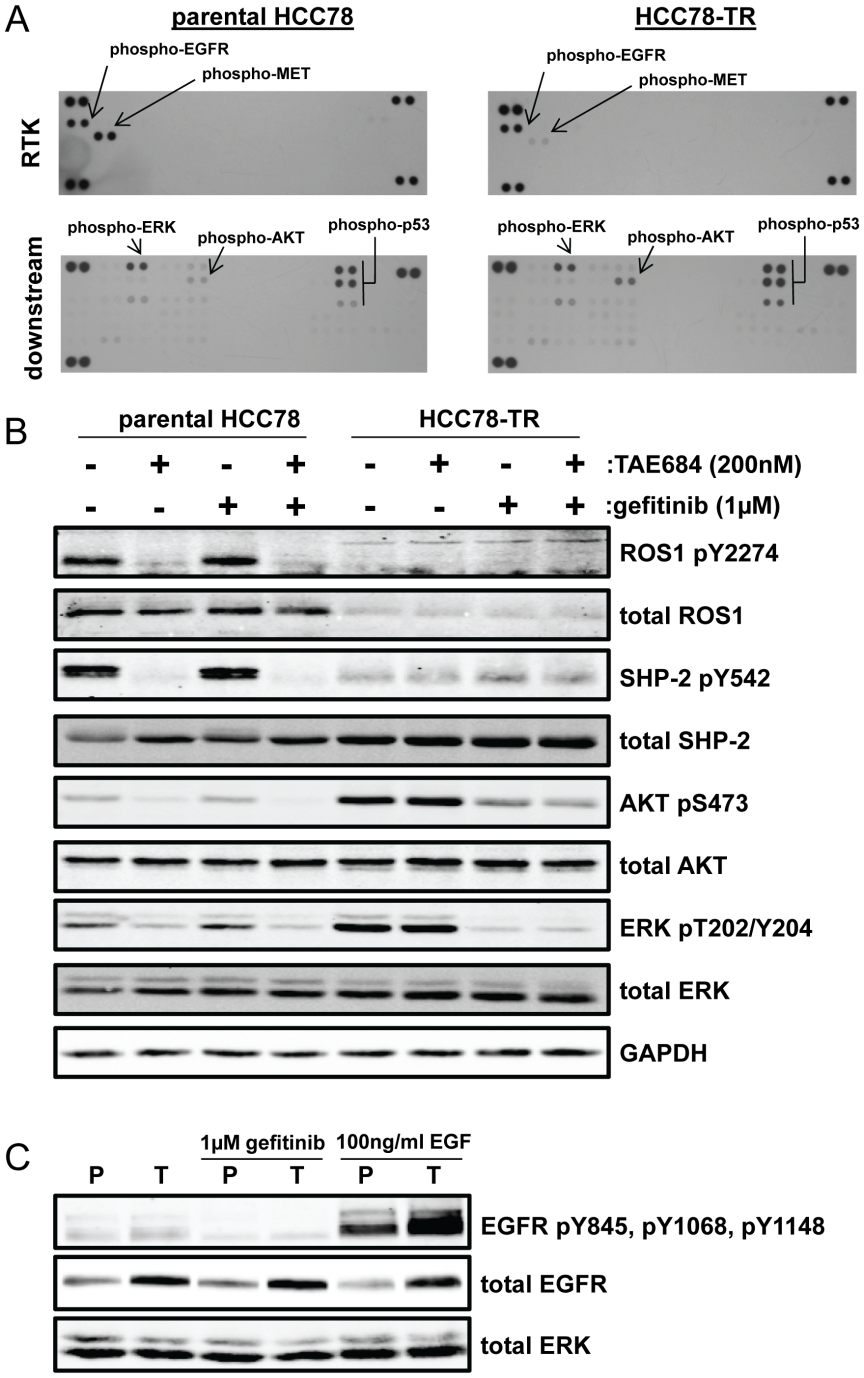
Growth and survival signaling pathway activation has become reliant on EGFR activity in the HCC78-TR cells. (A) Phospho-RTK (top) and phospho-kinase (bottom) array analyses performed on untreated parental HCC78 and HCC78-TR cells. Proteins of interest are labeled. Unlabeled spots at the corners of both sets of arrays are the positive control. (B) Parental HCC78 and HCC78-TR cells were treated with TAE684, gefitinib, or a combination of both for 4 hours. Lysates of the cells were then analyzed by Western blot using the indicated antibodies. (C) Parental HCC78 (P) and HCC78-TR (T) cells were left untreated, treated with 1****uM gefitinib for 4 hours, or treated with 100****ng/mL EGF for 10 minutes. Lysates of the cells were then analyzed by Western blot using the indicated antibodies.

The reduction in the amount of expressed ROS1 fusion protein, the persistence of basal AKT and ERK1/2 activation, and the lack of inhibition of AKT and ERK1/2 by the ROS1 inhibitors suggested that an alternative signaling pathway was being activated in the HCC78-TR cells. In an attempt to identify the upregulated pathway components, we performed two phospho-protein array experiments: one that examined receptor tyrosine kinases (RTKs) and one that analyzed downstream kinases (among other proteins). As we have observed previously, the parental HCC78 line expressed phosphorylated EGFR and MET when examined with the phospho-RTK array (Figure 5A) [28]. The HCC78-TR line still expressed phosphorylated EGFR, but phospho-MET was significantly reduced (Figure 5A). No other significantly phosphorylated receptor tyrosine kinases were observed. As predicted from western blot analysis, AKT phospho-S473 was increased in the HCC78-TR cells compared to the parental cells, as were several phosphorylation sites on p53 (Figure 5A). These were the only differences observed by phospho-protein array.

We have previously demonstrated that EGFR signaling is partially active in the parental HCC78 line, as a potent anti-proliferative effect of TAE684 could only be achieved with co-treatment with the EGFR inhibitor gefitinib [28]. Due to the observations that EGFR was the only significantly phosphorylated RTK in the HCC78-TR line, and that AKT, which is commonly activated downstream of EGFR, was more heavily phosphorylated in the HCC78-TR line, we hypothesized that perhaps EGFR signaling had been further engaged in the resistant cells. To test this hypothesis, we treated parental HCC78 and HCC78-TR cells with 200****nM TAE684, 1 µM gefitinib, or a combination of both for 4 hours. Again, phospho-AKT and phospho-ERK1/2 were sensitive to TAE684 treatment in the parental line but not the HCC78-TR line (Figure 5B). However, the situation was reversed when these lines were treated with gefitinib, with downstream signaling being sensitive in the HCC78-TR line but not the parental line (Figure 5B). Similar effects were observed with the chemically distinct EGFR inhibitors erlotinib, lapatinib, and afatinib, suggesting that the effects were due to on-target EGFR inhibition (Figure S6). Thus, WT EGFR had become the dominant driver of growth and survival signaling pathways in the HCC78-TR cells. This change in cellular signaling correlated with a modest increase in total EGFR levels in the HCC78-TR cells as compared to the parental HCC78 cells (Figure 5C). Autophosphorylation of EGFR, as determined by western blot using a cocktail of phosphorylation site-specific antibodies, was relatively low in both cell lines. However, upon stimulation with the EGFR ligand EGF, autophosphorylation was increased and was higher in the HCC78-TR cells (Figure 5C). mRNA quantitation revealed that most EGFR ligands were not significantly more expressed in the HCC78-TR cells, with the exception of NRG1 which displayed a 4-fold increase in mRNA levels (Figure S7).

**Figure 6:**
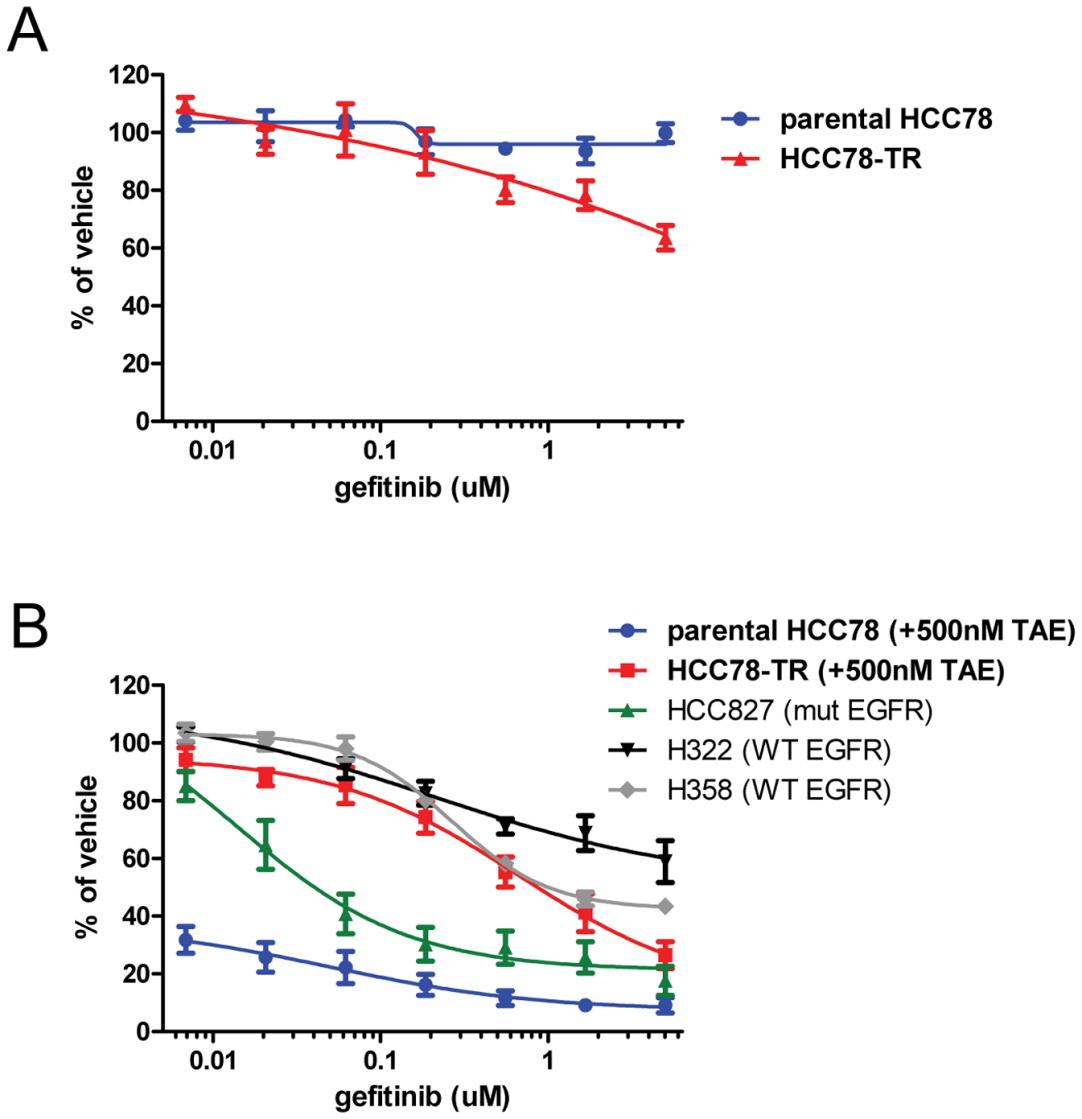
HCC78-TR cells have become sensitive to EGFR inhibition. (A) Parental HCC78 and HCC78-TR cells were treated with gefitinib as a single-agent for 3 days and then analyzed by MTS assay. (B) Parental HCC78 and HCC78-TR cells were co-treated with 500****nM TAE684 and gefitinib, and HCC827, H322, and H358 cells were treated with single-agent gefitinib for 3 days and then analyzed by MTS assay. Calculated IC_50_ values: parental HCC78 (below 50% with single-agent TAE684), HCC78-TR = 0.86 µM, HCC827 = 0.04 µM, H322 = >5 µM, and H358 = 1.0****uM. All values represent the mean ± SEM (n = 4).

Due to the switch in control of growth and survival signaling pathways from ROS1 to EGFR in the HCC78-TR cells, we hypothesized that proliferation of these cells would be sensitive to EGFR inhibition. To test this hypothesis, we treated both parental HCC78 and HCC78-TR cells with gefitinib as a single-agent in proliferation assays. As we have previously reported, gefitinib at concentrations up to 5 µM did not affect the proliferation of parental HCC78 cells (Figure 6A) [28]. However, the drug modestly inhibited the proliferation of HCC78-TR cells (Figure 6A). Similar effects were observed with erlotinib (data not shown). We then hypothesized that, since the HCC78-TR cells still expressed some, albeit reduced levels of the ROS1 fusion protein, a complete anti-proliferative effect induced by gefitinib would require co-inhibition of ROS1. To test this hypothesis, we treated HCC78-TR cells with a dose range of gefitinib in combination with 500****nM TAE684 (the concentration of drug that these cells were continuously cultured in). The addition of 500****nM TAE684 had no anti-proliferative effect on its own; however, it further sensitized the cells to gefitinib treatment (Figure 6B). Under these conditions, the HCC78-TR cells were not as sensitive as the HCC827 NSCLC cell line which is driven by E746_A750del EGFR. This is expected because activating mutations on EGFR enhance its affinity to EGFR kinase inhibitors [32]. However, the HCC78-TR cells were as or more sensitive than the WT EGFR expressing NSCLC lines H358 and H322, respectively (Figure 6B). These two cell lines have been reported to be highly sensitive to gefitinib when compared to other WT EGFR expressing NSCLC lines [33]. Importantly, in the HCC78-TR cells, significant anti-proliferative activity was observed at clinically relevant doses of gefitinib (∼1 µM) when combined with ROS1 inhibition [34].

**Figure 7:**
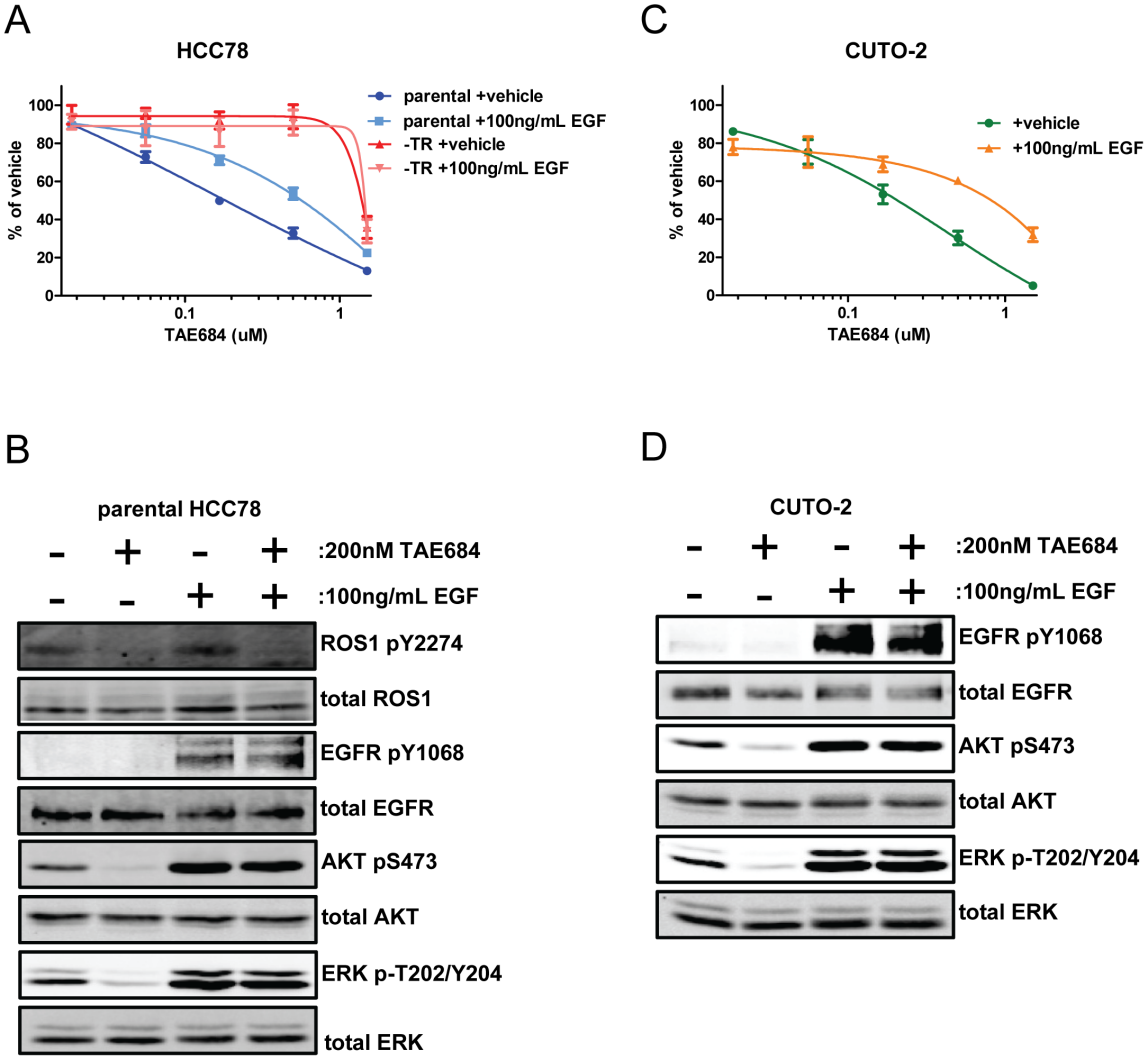
EGF stimulation desensitizes parental HCC78 cells and CUTO-2 cells to ROS1 inhibition. (A) Parental HCC78 and HCC78-TR cells were treated with TAE684 for 3 days with or without the addition of 100****ng/mL EGF and then analyzed by MTS assay. Values represent the mean ± SEM (n = 3). Calculated IC_50_ values for TAE684: parental+vehicle = 0.18 µM, parental+EGF = 0.57 µM, HCC78-TR+vehicle = 1.39 µM, and HCC78-TR+EGF = 1.45 µM. EGF significantly desensitized parental HCC78 but not HCC78-TR cells to TAE684 as determined by student’s paired t-test (p<0.05). (B) Parental HCC78 cells were treated with TAE684 for 4 hours, EGF for 10 minutes, or a combination of both. Lysates of the cells were then analyzed by Western blot using the indicated antibodies. (C) CUTO-2 cells were treated with TAE684 for 4 days with or without the addition of 100****ng/mL EGF and then analyzed by MTS assay. Values represent the mean ± SEM (n = 3). Calculated IC_50_ values for TAE684: +vehicle = 0.2 µM and +EGF = 0.81 µM. EGF significantly desensitized CUTO-2 cells to TAE684 as determined by student’s paired t-test (p<0.01). (D) CUTO-2 cells were treated with TAE684 for 4 hours, EGF for 10 minutes, or a combination of both. Lysates of the cells were then analyzed by Western blot using the indicated antibodies. Phosphorylated ROS1 bands were below the limit of detection and were therefore not included.

As a proof-of-concept that EGFR activation can de-sensitize ROS1 fusion-driven cells to ROS1 inhibition, we examined the effect of ligand-induced receptor activation on sensitivity. To accomplish this, we performed proliferation assays examining sensitivity to TAE684 in the absence or presence of the EGFR ligand EGF. We found that the addition of EGF at the beginning of the assay significantly desensitized the parental HCC78 cells to inhibition of ROS1 (Figure 7A). In contrast, EGF had no effect on the sensitivity of the HCC78-TR cells, which was expected due to the already maximal insensitivity of this line (*cf* Figure 2A). Mechanistically, the desensitization in the parental HCC78 cells correlated with a rescue by EGF from the TAE684-induced de-phosphorylation of AKT and ERK1/2 (Figure 7B).

A cell line was derived from the biopsy that was taken from the patient’s resistant tumor. Establishment of this cell line, which we have termed Colorado University Thoracic Oncology-2 (CUTO-2), took approximately one year in culture and was performed in the absence of a ROS1 inhibitor. When the cells reached sufficient passage number (>35 passages) and sufficient growth rate for experimental analysis, we examined ROS1 fusion gene status, ROS1 fusion protein expression, and sensitivity to ROS1 inhibition. The CUTO-2 cells were verified to still exhibit rearrangement of the *ROS1* gene and express a ROS1 fusion protein (Figure S8A, B). In proliferation assays, the CUTO-2 cells demonstrated a similar sensitivity to TAE684 and a slightly increased sensitivity to crizotinib compared to the parental HCC78 cells (Figure 7C and Figure S8C). Interestingly, like the parental HCC78 cells, sensitivity to ROS1 inhibition could be reduced by EGF application and this desensitization correlated with rescue from TAE684-induced reduction of phosphorylated AKT and ERK1/2 (Figure 7 C and D).

## Discussion

Acquired resistance to targeted therapies is a major limitation to the treatment of lung cancer patients. However, rational approaches to combat resistance can be developed once the molecular and cellular mechanisms that underlie it are identified. In this study, we investigated mechanisms of resistance to ROS1 inhibition in NSCLC. This was accomplished using tumor samples from a NSCLC patient who became resistant to crizotinib and a NSCLC cell line that we made resistant to ROS1 inhibition by continuous culture in the ROS1 inhibitor TAE684. Ideally, studies undertaken to examine resistance mechanisms involve sample banks from multiple patients and multiple different cell lines. However, as *ROS1* rearrangements are only present in 1–2% of NSCLC patients, we only had access to one patient who became resistant to treatment. Furthermore, prior to our derivation of the CUTO-2 line, HCC78 was the only published NSCLC cell line known to express a ROS1 fusion protein. Despite these limitations, the findings from this study have important implications for *ROS1* rearrangement-positive patients who become resistant to crizotinib treatment. Prior to our study, only one published report had identified a mechanism of resistance to ROS1 inhibition. In the study by Awad et al., a *ROS1* rearrangement-positive patient who initially responded to crizotinib but became resistant was found to have acquired a mutation at codon 2032 of a *ROS1* fusion gene (*CD74-ROS1*). This mutation was demonstrated to interfere with crizotinib binding to the ROS1 ATP-binding site [30]. Importantly, resistance-associated mutations in *ALK* have previously been found to co-exist with alternative pathway up-regulation, leaving open the possibility that this patient’s tumor cells had undergone additional changes that might have contributed to resistance [16].

We observed that neither the resistant patient sample nor the HCC78-TR cells had undergone *ROS1* kinase domain mutation, *ROS1* fusion gene amplification, EMT, or conversion to small cell lung cancer histology. These changes have been found to underlie resistance to EGFR inhibitors in *EGFR*-mutant NSCLC and to crizotinib in *ALK* rearrangement-positive NSCLC. However, in the HCC78-TR cells, we found that ROS1 fusion protein levels were reduced and growth and survival signaling pathways switched from being primarily dependent on ROS1 activity to being primarily dependent on EGFR activity. The mechanism behind this switch remains unclear, although it occurred in the absence of a significant increase in EGFR autophosphorylation, suggesting that an autocrine signaling mechanism was not responsible. It is noteworthy that EGFR kinase activity is not always dependent on autophosphorylation and thus low levels of EGFR phosphorylation do not preclude its signaling activity [35]. Hypothetically, a ‘re-wiring’ of cellular signaling networks created an enhanced dependence on EGFR activity to activate growth and survival signaling cascades, however this hypothesis requires further experimentation. Regardless of the specific mechanism, the signaling switch resulted in the proliferation of the HCC78-TR cells becoming partially sensitive to EGFR inhibition alone. Upon co-inhibition of ROS1, this cell line became as or more sensitive than other WT EGFR expressing NSCLC lines that have been reported to be very sensitive to gefitinib [33]. Importantly, clinically relevant doses of gefitinib (∼1 µM) were effective at inhibiting downstream signaling and inducing an anti-proliferative effect [34]. While EGFR inhibitors are generally thought to be efficacious primarily in lung cancers expressing *EGFR* with activating mutations, a substantial number of patients who are negative for these mutations derive some modest benefit from treatment, suggesting that WT *EGFR* can support tumor growth [36]. Furthermore, activity of EGFR inhibitors in NSCLC cells is correlated with the ability of the drugs to reduce AKT and ERK1/2 activation, and single-agent EGFR inhibition resulted in de-phosphorylation of these proteins in our HCC78-TR cells (Figures 5B and S6) [37].

Activation of WT EGFR signaling has also been described as a mechanism of resistance to ALK inhibition in *ALK* rearrangement-positive NSCLC. This has been observed in cases of primary resistance of cell lines to ALK inhibition, in which cells that express highly phosphorylated EGFR and/or ERBB2 require co-inhibition of these proteins with ALK to achieve downstream signaling inactivation and a potent anti-proliferative effect [25], [38]. EGFR pathway activation has also been observed in cell line models of acquired resistance, and this effect has been demonstrated to correlate with increased expression of EGFR ligands [15], [16]. Again, in these cases, co-inhibition of EGFR and ALK is required for ERK1/2 and AKT de-phosphorylation and full inhibition of proliferation. Furthermore, increased phosphorylation of WT EGFR has been demonstrated in biopsies from *ALK*-positive patients who have become resistant to crizotinib, as compared to pre-treatment samples [16]. In *ALK* rearrangement-positive cells that are sensitive to ALK inhibition, application of EGF reduces sensitivity in terms of downstream signaling activation and proliferation [15], [26], [38], [39]. Interestingly, EGFR pathway activation as a resistance mechanism is not limited to the WT receptor, as activating *EGFR* mutations have been observed in some crizotinib-resistant patients [14], [40].

The lack of an identified genetic mechanism underlying the switch to EGFR-dependent cellular signaling in the resistant cells precluded direct examination of the patient samples for a similar effect. However, in lieu of this, we examined a cell-line derived from the patient’s biopsy taken at the time of resistance. This cell line, CUTO-2, was found to be sensitive to ROS1 inhibition. However, the cells appeared to be ‘primed’ to engage the EGFR pathway, as exposure to EGF reduced sensitivity to TAE684 both in terms of proliferation and downstream signaling, effects that were mirrored in the parental HCC78 cells (Figure 7). While this does not prove that EGFR pathway engagement was the mechanism of resistance in this patient, it does suggest that EGFR activity was sufficient to induce resistance to crizotinib in the patient’s tumor cells. Importantly, this appears to be a common, although not universal, effect in cell lines driven by other oncogenes [26].

The results from this study, coupled with similar findings that have been reported in *ALK* rearrangement-positive NSCLC, suggest that EGFR pathway activation may be a common mechanism of resistance to ROS1 inhibition. EGFR inhibitors are already approved by regulatory agencies throughout the world. Therefore, the combination of crizotinib (or other ROS1 inhibitors) with EGFR inhibition may be an effective strategy to combat resistance in some patients. Furthermore, it is possible that, by taking away a primary mechanism of resistance, this combination strategy may delay resistance if initiated as soon as that patient tests positive for *ROS1* rearrangement.

## Materials and Methods

### Patient Samples

Written informed consent was obtained from the patient prior to analyses of the patient’s tumor sample. The consent form and protocol was reviewed and approved by the Colorado Multiple Institutional Review Board.

### Cell Lines and Reagents

HCC78, H322, HCC4006, HCC827, and H358 were obtained from John D. Minna and used as previously described [28], [33], [41], [42]. NVP-TAE684, crizotinib (PF-02341066), gefitinib, and erlotinib were purchased from Selleck Chemicals (Houston, TX). Antibodies used were as follows: ROS1 pY2274 (3078, Cell Signaling, Danvers, MA), total ROS1 (sc-6347, Santa Cruz Biotechnology, Santa Cruz, CA), SHP-2 pY542 (3751, Cell Signaling), total SHP-2 (610621, BD Biosciences), AKT pS437 (4058, Cell Signaling), total AKT (2920, Cell Signaling), ERK pT202/Y204 (9101, Cell Signaling), total ERK (9107, Cell Signaling), EGFR pY845 (2231, Cell Signaling), EGFR pY1068 (2234, Cell Signaling), EGFR pY1148 (4404, Cell Signaling), total EGFR (2232, Cell Signaling), and GAPDH (MAB274, Millipore).

### Derivation of Cell Lines

The HCC78-TR cell line was derived by continuous culture in TAE684 (gradually increasing doses until the cells were able to proliferate normally in a 500****nM concentration). Once established, the HCC78-TR cells were continuously cultured in 500****nM TAE684. Ten subclones were made from this cell line, but all were found to all be equally sensitive to gefitinib and express equivalently phosphorylated levels of AKT, suggesting that all had undergone the primary changes observed in the pooled population (data not shown). Derivation of the CUTO-2 cell line was performed following written consent by the patient and approval by the Colorado Multiple Institutional Review Board (COMIRB). The line was derived from a sample of the patient’s surgically resected tissue that was disaggregated using the ‘mechanical spill-out method’ in order to obtain tumor aggregates free of stromal components [43]. Cell aggregates were plated out onto a 25****cm flask and cultured in serum free ACL4 media to discourage outgrowth of normal stromal cells. Once the tumor cells became the predominately established cell type in the culture flask, the culture was subjected to differential trypsinization in order to dislodge the remaining minor population of stromal cells. After this enrichment process, tumors cells were cultured in ACL4 media supplemented with 5% heat inactivated fetal bovine serum and routinely passaged using this media. The established CUTO-2 cell line was later adapted to grow in RPMI1640 with 10% FBS for ease of culturing and experimentation.

### RNA Isolation and Sequencing

RNA isolation from patient samples was performed as previously reported [28]. RNA isolation from cell lines was performed using the RNeasy kit from Qiagen per the manufacturer’s instructions. Standard Sanger sequencing techniques were performed to sequence the *ROS1* kinase domain in the patient samples and cell lines as well as *EGFR* and *KRAS* in the cell lines. Mutational status of these genes (along with other common oncogenes) in the cell lines was then confirmed by RNA-seq analysis (see below). The *EGFR*, *KRAS,* and *BRAF* status of the patient samples was determined by SNaPshot analysis (Applied Biosystems).

### Cellular Proliferation

MTS assays and data analysis were performed as previously described [28]. CUTO-2 cells were analyzed 4 days after treatment (instead of 3 days for the other cell lines) due to a slower growth rate.

### Fluorescence In-Situ Hybridization

Break-apart FISH analysis was designed and performed as previously described [28].

### Immunoblotting and Phospho-Arrays

Immunoblotting was performed as previously described [28]. Phospho-RTK Array Kit (ARY001) and Phospho-Kinase Array Kit (ARY003) from R&D Systems were performed per the manufacturer’s instructions.

### mRNA Quantitation

High-throughput mRNA sequencing (RNA-seq) of each sample (two samples per cell line) was obtained from the Illumina HiSeq2000. On average, approximately 50 million (coverage ranged from 45 to 55 million reads) paired-end 100 bp sequencing reads were obtained per sample. To analyze the RNA-seq data, the reads were mapped against the human genome using Tophat (version 2.0.5) (PMID: 19289445). NCBI reference annotation (build 37.2) was used as a guide, and allowing 3 mismatches for the initial alignment and 2 mismatches per segment with 25 bp segments. On average, 95% of the reads aligned to the human genome. Transcripts were assembled using Cufflinks (version 2.0.2) (PMID: 20436464) to assemble the transcripts using the RefSeq annotation as the guide, but allowing for novel isoform discovery in each sample. The data were fragment bias corrected, multi-read corrected, and normalized by the total number of reads. Differentially expressed genes were identified by Cuffdiff after merging the transcript assemblies. All other analyses were performed in R/Bioconductor (R version 2.14.1 (2011-12-22)).

### Lentiviral Constructs and Transduction

We have previously described the creation of the SDC4:ROS1 construct, and transduction was performed as previously described [28].

## Supporting Information

Figure S1 **Resistant tumor cells did not undergo EMT.** Vimentin IHC staining of post-resistance tumor biopsy. Tumor cells did not demonstrate significant staining. However, supporting stromal cells within the same slide did stain positive, suggesting that the staining was successful.
(PDF)

Figure S2 **HCC78-TR cells remain resistant to ROS1 inhibition when cultured without 500 nM TAE684.** HCC78-TR cells were cultured without TAE684 for up to 6 months and 47 passages. Cells (passage numbers 39–47) were treated with TAE684 for 3 days and then analyzed by MTS assay. Values represent the mean ± SEM (n = 3). Calculated IC_50_ value for TAE684 = 1.3 µM.
(PDF)

Figure S3 ***SLC34A2-ROS1*** **fusion gene mRNA levels are reduced in the HCC78-TR cells compared to the parental HCC78 cells.** Transcript levels of the *SLC34A2-ROS1* fusion gene as measured by RNA-seq analysis. Data (number of individual reads supporting the specific splicing variant) is an average of 2 independent samples for each cell line. Splicing variants are as follows: SLC4;R32 = fusion of *SLC34A2* exon 4 to *ROS1* exon 32, SLC4;R33 = fusion of *SLC34A2* exon 4 to *ROS1* exon 33, and SLC4;R34 = fusion of *SLC34A2* exon 4 to *ROS1* exon 34. Note that the SLC4;R33 variant has not been previously reported in this cell line and its existence requires further validation.
(PDF)

Figure S4 **Introduction of an activated** ***ROS1*** **fusion gene into HCC78-TR cells does not lead to re-sensitization to ROS1 inhibition.** HCC78-TR cells were transduced with empty vector (and cultured in 500****nM TAE684) or SDC4-ROS1 (and cultured with or without 500****nM TAE684). Cells were then treated with TAE684 for 3 days and then analyzed by MTS assay. Values represent the mean ± SEM (n = 3). No significant differences were observed.
(PDF)

Figure S5 **mRNA quantitation reveals that EMT has not occurred in the HCC78-TR cells.** *CDH1* (left) and *VIM* (right) levels in parental HCC78 and HCC78-TR cells as measured by RNA-seq analysis. Data (FPKM, Fragments Per Kilobase of transcript per Million mapped reads) is an average of 2 independent samples.
(PDF)

Figure S6 **Four chemically distinct EGFR inhibitors all reduce AKT and ERK activation in HCC78-TR cells but not parental HCC78 cells.** Parental HCC78 (top) or HCC78-TR (bottom) cells were treated with the indicated drugs for 4 hours. Lysates of the cells were then analyzed by Western blot using the indicated antibodies.
(PDF)

Figure S7 **EGFR ligand expression, with the exception of NRG1, is not increased in the HCC78-TR cells.** EGFR ligand levels in parental HCC78 and HCC78-TR cells as measured by RNA-seq analysis. Data (FPKM, Fragments Per Kilobase of transcript per Million mapped reads) is an average of 2 independent samples.
(PDF)

Figure S8 **CUTO-2 cells retain the rearranged** ***ROS1*** **gene, express a ROS1 fusion protein, and are sensitive to crizotinib.** (A) Break-apart FISH analysis of CUTO-2 cells. Red probes are to the 5′ region of *ROS1* and green probes to the 3′ region. (B) Western blot analysis of CUTO-2 lysates probed with an antibody specific to total ROS1. (C) CUTO-2 cells were treated with crizotinib for 4 days and then analyzed by MTS assay. Values represent the mean ± SEM (n = 3). Calculated IC_50_ value for crizotinib = 0.38 µM.
(PDF)

Table S1 (PDF)
