# Supplementary figures and images for: Resistance to ROS1 Inhibition Mediated by EGFR Pathway Activation in Non-Small Cell Lung Cancer
Source: PLoS One. 2013 Dec 13;8(12):e82236. doi: 10.1371/journal.pone.0082236 (PMC3862576; doi:10.1371/journal.pone.0082236)

Figure S1

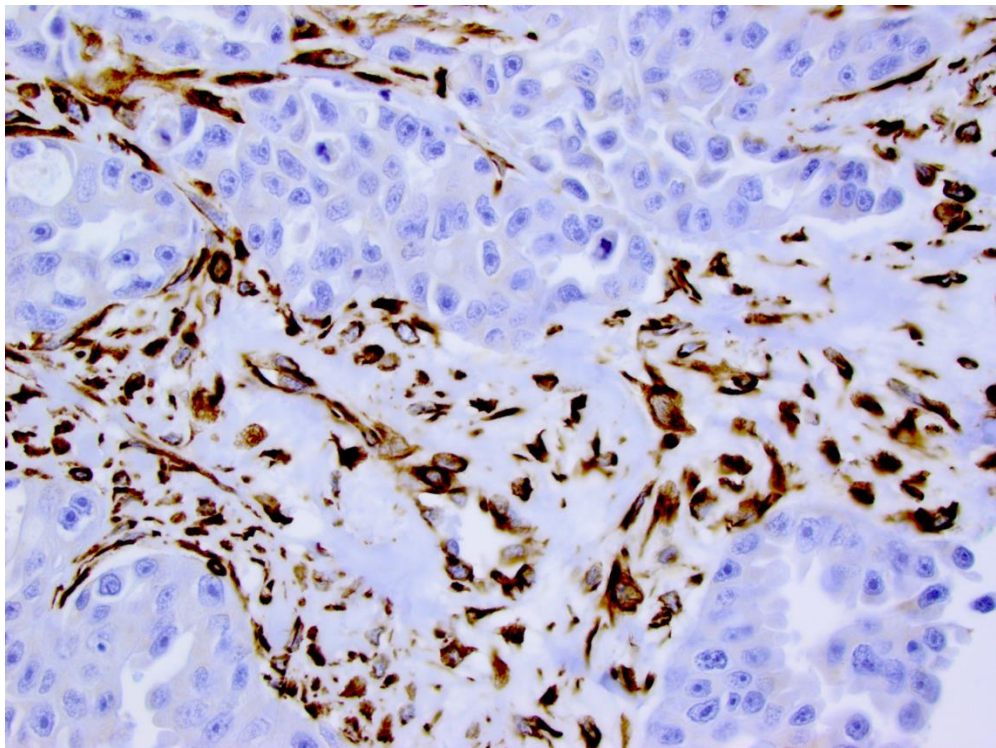

Supplement: Figure S1 — Resistant tumor cells did not undergo EMT. Vimentin IHC staining of post-resistance tumor biopsy. Tumor cells did not demonstrate significant staining. However, supporting stromal cells within the same slide did stain positive, suggesting that the staining was successful. (PDF) [file pone.0082236.s001.pdf]

Figure S2

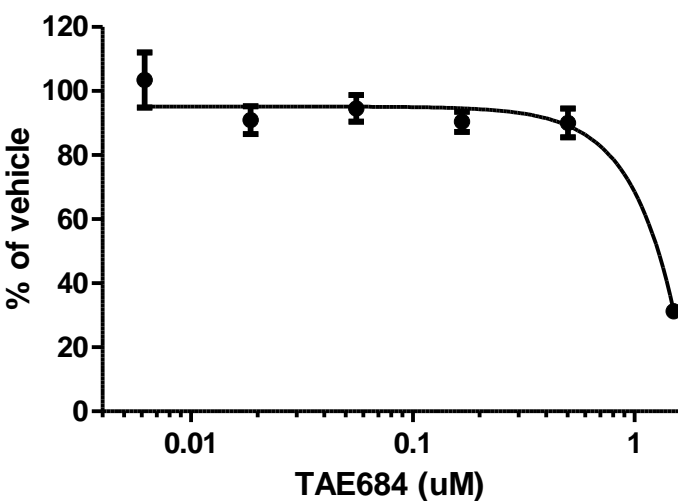

Supplement: Figure S2 — HCC78-TR cells remain resistant to ROS1 inhibition when cultured without 500 nM TAE684. HCC78-TR cells were cultured without TAE684 for up to 6 months and 47 passages. Cells (passage numbers 39–47) were treated with TAE684 for 3 days and then analyzed by MTS assay. Values represent the mean ± SEM (n = 3). Calculated IC50 value for TAE684 = 1.3 µM. (PDF) [file pone.0082236.s002.pdf]

Figure S3

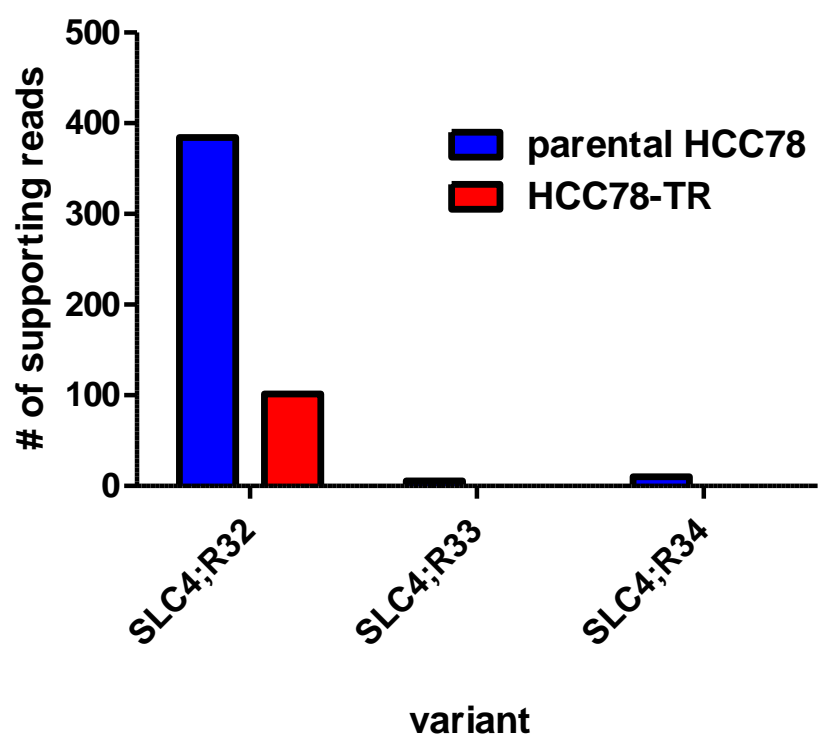

Supplement: Figure S3 — SLC34A2-ROS1 fusion gene mRNA levels are reduced in the HCC78-TR cells compared to the parental HCC78 cells. Transcript levels of the SLC34A2-ROS1 fusion gene as measured by RNA-seq analysis. Data (number of individual reads supporting the specific splicing variant) is an average of 2 independent samples for each cell line. Splicing variants are as follows: SLC4;R32 = fusion of SLC34A2 exon 4 to ROS1 exon 32, SLC4;R33 = fusion of SLC34A2 exon 4 to ROS1 exon 33, and SLC4;R34 = fusion of SLC34A2 exon 4 to ROS1 exon 34. Note that the SLC4;R33 variant has not been previously reported in this cell line and its existence requires further validation. (PDF) [file pone.0082236.s003.pdf]

Figure S4

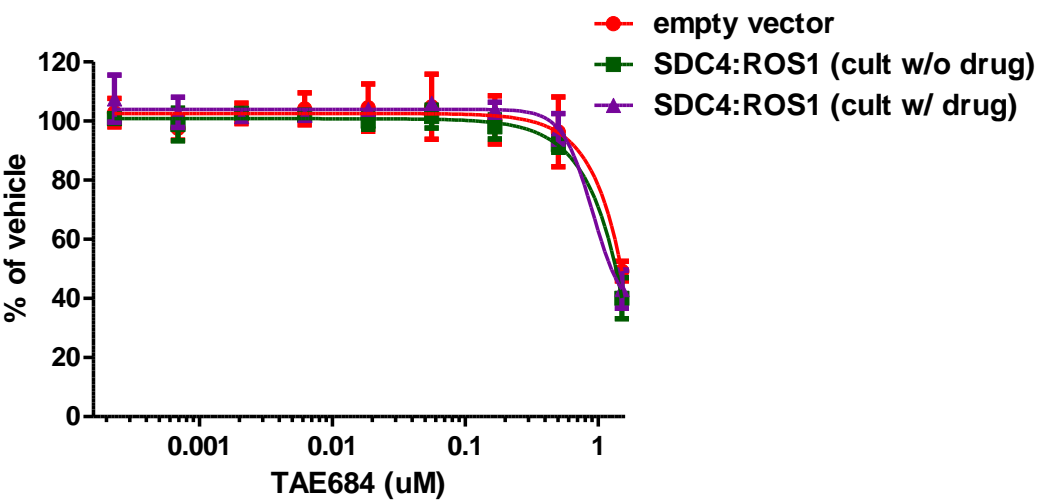

Supplement: Figure S4 — Introduction of an activated ROS1 fusion gene into HCC78-TR cells does not lead to re-sensitization to ROS1 inhibition. HCC78-TR cells were transduced with empty vector (and cultured in 500nM TAE684) or SDC4-ROS1 (and cultured with or without 500nM TAE684). Cells were then treated with TAE684 for 3 days and then analyzed by MTS assay. Values represent the mean ± SEM (n = 3). No significant differences were observed. (PDF) [file pone.0082236.s004.pdf]

Figure S5

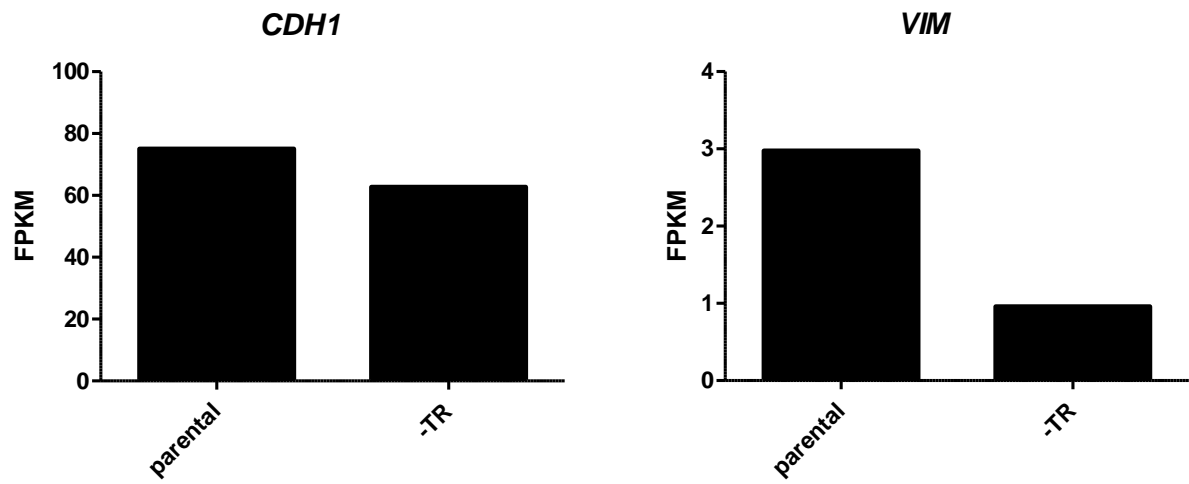

Supplement: Figure S5 — mRNA quantitation reveals that EMT has not occurred in the HCC78-TR cells. CDH1 (left) and VIM (right) levels in parental HCC78 and HCC78-TR cells as measured by RNA-seq analysis. Data (FPKM, Fragments Per Kilobase of transcript per Million mapped reads) is an average of 2 independent samples. (PDF) [file pone.0082236.s005.pdf]

Figure S6

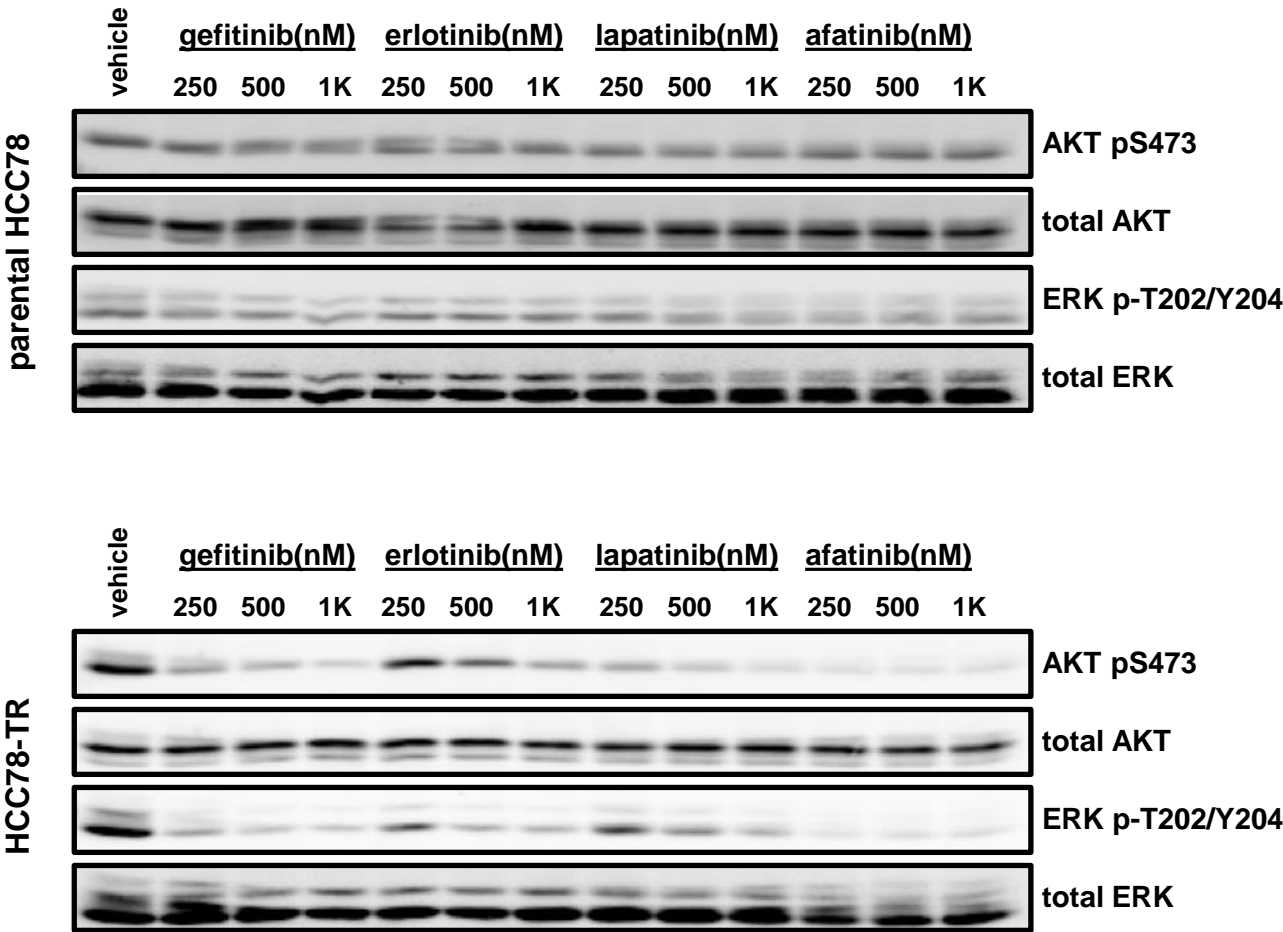

Supplement: Figure S6 — Four chemically distinct EGFR inhibitors all reduce AKT and ERK activation in HCC78-TR cells but not parental HCC78 cells. Parental HCC78 (top) or HCC78-TR (bottom) cells were treated with the indicated drugs for 4 hours. Lysates of the cells were then analyzed by Western blot using the indicated antibodies. (PDF) [file pone.0082236.s006.pdf]

Figure S7

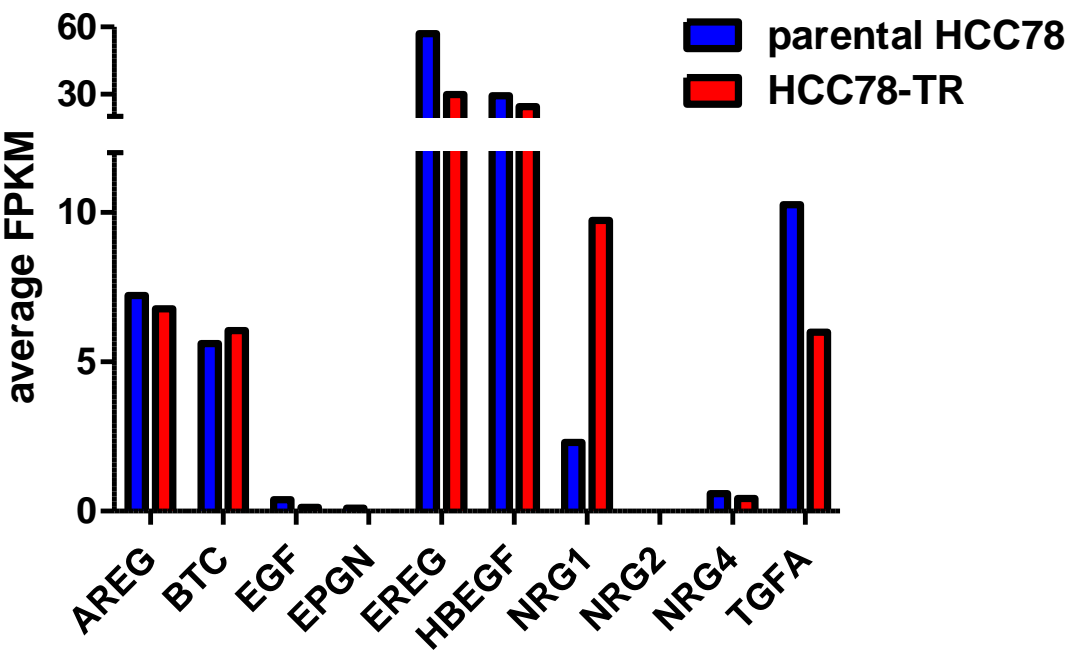

Supplement: Figure S7 — EGFR ligand expression, with the exception of NRG1, is not increased in the HCC78-TR cells. EGFR ligand levels in parental HCC78 and HCC78-TR cells as measured by RNA-seq analysis. Data (FPKM, Fragments Per Kilobase of transcript per Million mapped reads) is an average of 2 independent samples. (PDF) [file pone.0082236.s007.pdf]

Figure S8

A

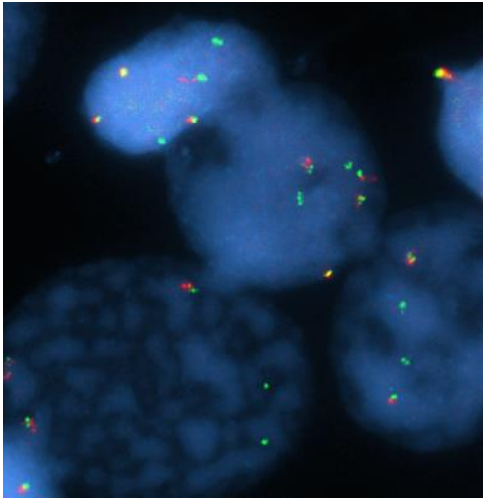

B

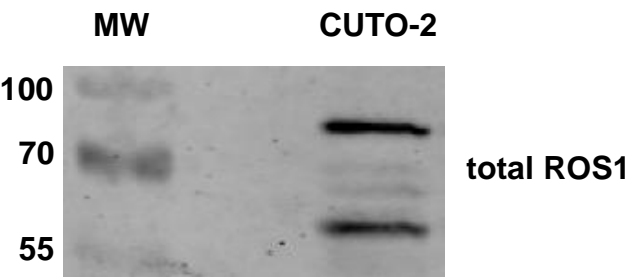

C

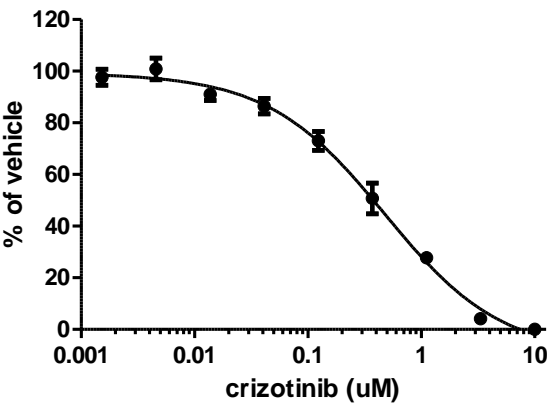

Supplement: Figure S8 — CUTO-2 cells retain the rearranged ROS1 gene, express a ROS1 fusion protein, and are sensitive to crizotinib. (A) Break-apart FISH analysis of CUTO-2 cells. Red probes are to the 5′ region of ROS1 and green probes to the 3′ region. (B) Western blot analysis of CUTO-2 lysates probed with an antibody specific to total ROS1. (C) CUTO-2 cells were treated with crizotinib for 4 days and then analyzed by MTS assay. Values represent the mean ± SEM (n = 3). Calculated IC50 value for crizotinib = 0.38 µM. (PDF) [file pone.0082236.s008.pdf]
