# Supplementary material for: Resistance to ROS1 Inhibition Mediated by EGFR Pathway Activation in Non-Small Cell Lung Cancer
Source: PLoS One. 2013 Dec 13;8(12):e82236. doi: 10.1371/journal.pone.0082236 (PMC3862576; doi:10.1371/journal.pone.0082236)
Supplement: Table S1 — (PDF) [file pone.0082236.s009.pdf]

Table S1

gene	description	Parental HCC78 avg FPKM	HCC78-TR avg FPKM	% change
ABCA1	ATP-binding cassette, sub-family A (ABC1), member 1	0.40	0.16	-59.2
ABCA12	ATP-binding cassette, sub-family A (ABC1), member 12	1.12	0.83	-25.8
ABCA2	ATP-binding cassette, sub-family A (ABC1), member 2	1.87	1.60	-14.8
ABCA3	ATP-binding cassette, sub-family A (ABC1), member 3	2.79	2.94	5.2
ABCA7	ATP-binding cassette, sub-family A (ABC1), member 7	5.17	5.91	14.2
ABCB10	ATP-binding cassette, sub-family B (MDR/TAP), member 10	15.09	9.94	-34.1
ABCB6	ATP-binding cassette, sub-family B (MDR/TAP), member 6	2.55	2.05	-19.8
ABCB7	ATP-binding cassette, sub-family B (MDR/TAP), member 7	13.82	14.46	4.7
ABCB8	ATP-binding cassette, sub-family B (MDR/TAP), member 8	10.85	19.23	77.2
ABCC1	ATP-binding cassette, sub-family C (CFTR/MRP), member 1	26.40	30.49	15.5
ABCC10	ATP-binding cassette, sub-family C (CFTR/MRP), member 10	3.63	3.59	-1.1
ABCC13	ATP-binding cassette, sub-family C (CFTR/MRP), member 13, pseudogene	0.12	0.04	-68.7
ABCC2	ATP-binding cassette, sub-family C (CFTR/MRP), member 2	0.21	0.17	-20.2
ABCC3	ATP-binding cassette, sub-family C (CFTR/MRP), member 3	15.75	7.46	-52.6
ABCC4	ATP-binding cassette, sub-family C (CFTR/MRP), member 4	8.95	14.42	61.0
ABCC5	ATP-binding cassette, sub-family C (CFTR/MRP), member 5	7.24	8.42	16.3
ABCC6	ATP-binding cassette, sub-family C (CFTR/MRP), member 6	0.59	0.94	58.4
ABCC6P2	ATP-binding cassette, sub-family C, member 6 pseudogene 2	0.24	0.32	33.1
ABCC9	ATP-binding cassette, sub-family C (CFTR/MRP), member 9	0.72	0.62	-13.6
ABCD1	ATP-binding cassette, sub-family D (ALD), member 1	1.83	1.13	-38.3
ABCD3	ATP-binding cassette, sub-family D (ALD), member 3	22.91	22.83	-0.4
ABCD4	ATP-binding cassette, sub-family D (ALD), member 4	9.78	10.26	4.9
ABCE1	ATP-binding cassette, sub-family E (OABP), member 1	69.55	53.84	-22.6
ABCF1	ATP-binding cassette, sub-family F (GCN20), member 1	29.31	35.52	21.2
ABCF2	ATP-binding cassette, sub-family F (GCN20), member 2	33.00	44.49	34.8
ABCF3	ATP-binding cassette, sub-family F (GCN20), member 3	22.43	24.07	7.3

The above data represents mRNA expression values obtained from a next generation sequencing analysis of the transcriptomes of both parental HCC78 and HCC78-TR cells. Data is average FPKM value (average of 2 independent samples) and the % change of the HCC78-TR cells as compared to the parental HCC78s. Only genes in which at least one FPKM value was greater than 0.1 are included.
